# Capsule-Targeting Depolymerase, Derived from *Klebsiella* KP36 Phage, as a Tool for the Development of Anti-Virulent Strategy

**DOI:** 10.3390/v8120324

**Published:** 2016-12-01

**Authors:** Grażyna Majkowska-Skrobek, Agnieszka Łątka, Rita Berisio, Barbara Maciejewska, Flavia Squeglia, Maria Romano, Rob Lavigne, Carsten Struve, Zuzanna Drulis-Kawa

**Affiliations:** 1 Institute of Genetics and Microbiology, University of Wroclaw, Przybyszewskiego 63/77, 51-148 Wroclaw, Poland; agnieszkalatka1989@gmail.com (A.Ł.); barbara-boczkowska@wp.pl (B.M.); zuzanna.drulis-kawa@uwr.edu.pl (Z.D.-K.); 2 Institute of Biostructures and Bioimaging, National Research Council, Via Mezzocannone 16, I-80134 Naples, Italy; rita.berisio@unina.it (R.B.); squegliaflavia@gmail.com (F.S.); romanom87@hotmail.it (M.R.); 3 Laboratory of Gene Technology, KU Leuven, Kasteelpark Arenberg 21, box 2462, B-3001 Leuven, Belgium; rob.lavigne@kuleuven.be; 4 Department of Microbiology and Infection Control, Statens Serum Institut, Artillerivej 5, DK-2300S Copenhagen, Denmark; CAS@ssi.dk; 5 World Health Organization Collaborating Centre for Reference and Research on Escherichia and Klebsiella, Statens Serum Institut, Artillerivej 5, DK-2300S Copenhagen, Denmark

**Keywords:** bacteriophage, *Klebsiella* sp., capsule, polysaccharide depolymerase

## Abstract

The rise of antibiotic-resistant *Klebsiella pneumoniae*, a leading nosocomial pathogen, prompts the need for alternative therapies. We have identified and characterized a novel depolymerase enzyme encoded by *Klebsiella* phage KP36 (depoKP36), from the *Siphoviridae* family. To gain insights into the catalytic and structural features of depoKP36, we have recombinantly produced this protein of 93.4 kDa and showed that it is able to hydrolyze a crude exopolysaccharide of a *K. pneumoniae* host. Using in vitro and in vivo assays, we found that depoKP36 was also effective against a native capsule of clinical *K. pneumoniae* strains, representing the K63 type, and significantly inhibited *Klebsiella*-induced mortality of *Galleria mellonella* larvae in a time-dependent manner. DepoKP36 did not affect the antibiotic susceptibility of *Klebsiella* strains. The activity of this enzyme was retained in a broad range of pH values (4.0–7.0) and temperatures (up to 45 °C). Consistently, the circular dichroism (CD) spectroscopy revealed a highly stability with melting transition temperature (T*m*) = 65 °C. In contrast to other phage tailspike proteins, this enzyme was susceptible to sodium dodecyl sulfate (SDS) denaturation and proteolytic cleavage. The structural studies in solution showed a trimeric arrangement with a high β-sheet content. Our findings identify depoKP36 as a suitable candidate for the development of new treatments for *K. pneumoniae* infections.

## 1. Introduction

*Klebsiella pneumoniae* ranks among the eight most common etiological factors of nosocomial infections, including pneumonia and urinary tract infections, accounting for up to 8% of all cases [1]. Furthermore, it is the second cause (after *Escherichia coli*) of both hospital- and community-acquired gram-negative bacteremia [1]. It has been reported that the number of *Klebsiella* strains harbouring extended spectrum beta-lactamases (ESBLs) and carbapenemases (KPC, MBL, OXA-48), which confer multidrug resistance, has significantly increased in recent years [2,3,4,5]. In Europe, the prevalence of ESBL- and KPC-producing *Klebsiella* among invasive isolates has reached 21% and 8.3%, respectively.

One of the most important virulence factors of *K. pneumoniae* is the polysaccharide, that surrounds the cell and may be organized into a distinct structure termed a capsule (capsular polysaccharide, CPS), or it can be released as an exopolysaccharide (EPS). CPS is a complex of acidic polysaccharide structure composed of repeating subunits of three to six sugars [1], with numerous configurations leading to identify at least 78 distinct CPS types [6,7,8]. CPS is critical for the resistance of *K. pneumoniae* to host defense mechanisms, suppression of early inflammatory response, adherence, and biofilm formation [9]. It also impedes or blocks the penetration of certain antibiotics into bacterial cells [10]. Additionally, it can provide a physical barrier to prevent the harmful effects of the environmental conditions, and to limit or preclude phage access [11]. The co-evolution of phages and their host bacteria has resulted in the formation of phage-encoded enzymes that depolymerize the highly specific polysaccharide structures, in order to gain access to the outer membrane and subsequently inject DNA into the bacterium [12,13]. The depolymerase enzymes associated with capsular-targeting phage particles reduce the viscosity of CPS and strip it from the surface of encapsulated bacteria [12]. The majority of *Klebsiella* acting enzymes randomly attack glycosidic linkages of CPS to release repeating units of the polymer [14,15,16]. The enzymatic loss of a capsule or alteration of its composition, makes bacteria devoid of an important shield and, therefore, more susceptible. As a result, capsular-targeting phages or their depolymerases in combination with other therapeutic agents could be exploited for the eradication of virulent pathogens.

Multiple applications of depolymerases have been proposed, including determination of *Klebsiella* capsular types for clinical strains [8,17], production of oligosaccharides from polysaccharides [18,19], or as alternative therapeutic agents to treat *Klebsiella* infections [17,20]. The latter application is of particular importance, as this opportunistic pathogen is a growing concern for public health.

In our previous study, the phage KP36 (vB_KpnS_KP36), whose genome encompasses 49,797 base pairs (bp) encoding 80 gene products, was characterized [21]. Transmission electron microscopy and genome sequence analyses revealed that this phage belongs to the family *Siphoviridae*, and is currently classified as a member of the “*KP36likevirus*” genus within subfamily *Tunavirinae* [22].

In the present work, we proved that the *K. pneumoniae* phage KP36 expresses an enzyme able to degrade the bacterial capsule and that this activity is conditioned by a tailspike protein with depolymerase activity. We also recombinantly produced this protein and characterized its biochemical properties, structural features in solution, as well as its activity in vitro and in vivo on *Galleria mellonella* model.

## 2. Materials and Methods

### 2.1. Phage, Bacterial Strains, and Culture Conditions

We originally isolated *Klebsiella* phage KP36 from biologically-treated sewage in Poland using the *K. pneumoniae* clinical isolate 486 as the host strain [21]. This phage and bacterial strain have been deposited in the Polish Collection of Microorganisms (Institute of Immunology and Experimental Therapy, Polish Academy of Sciences, Wroclaw, Poland) under accession numbers F/00068 and B/F/00068, respectively. They are publicly available from the authors upon request. The phage genome sequence has been deposited in GenBank/EMBL under the accession NC_029099. Besides the host strain, ESBL-producing clinical isolates were also analyzed using the spot method. These included the host strain, nine other strains (11, 27, 39, 74, 77, 271, 767, 1636, 7809131) belonging to the Institute of Genetics and Microbiology Bacterial Collection (Wroclaw, Poland), and *K. pneumoniae* 700603 obtained from the American Type Culture Collection (Microbiologics, St. Cloud, MN, USA). The evaluation of capsular type was performed using wzi sequencing according to the method of Brisse et al. [23]. *Klebsiella* strains were routinely grown in Trypticase Soy Broth or Agar (TSB or TSA, BioMérieux, Marcy-l’Étoile, France) at 37 °C. *E. coli* strains (Invitrogen, Thermo Fisher Scientific, Waltham, MA, USA) were used: top 10 F′ for plasmid propagation and BL21(DE3) for recombinant protein expression. These strains were cultivated in Lysogeny Broth (LB, BioCorp, Warszawa, Poland) at 37 °C.

### 2.2. Cloning, Expression, and Purification

Bacteriophage KP36 genomic DNA was extracted and purified as previously described [21]. The *gp50* open reading frame (ORF; NCBI acc. No. YP_009226010.1) encoding the depolymerase enzyme encoded by *Klebsiella* phage KP36 (depoKP36) was amplified by PCR using the specific primer pair: 5′-ATGGGGATTAAAACGCGGGTTACATTC-3′ and 5′-TTTCAGATCCTTAATGCATAGTTATATT C-3′ (Genomed, Warszawa, Poland) and Pfu DNA polymerase (Fermentas, Thermo Fisher Scientific). The 2649 bp PCR amplification product was subsequently cloned into the pEXP5-CT/TOPO^®^ expression vector (Invitrogen, Carlsbad, CA, USA) with a C-terminal His6 tag. Clone integrity was verified by DNA sequencing (Genomed). Following transformation of the correct construct into *E. coli* BL21(DE3) strain, plasmid-bearing cells were grown in 750 mL of LB supplemented with 100 µg/mL ampicillin at 37 °C with vigorous agitation to an optical density (OD_600nm_) of ~0.6. Recombinant protein expression was induced at 20 °C for 18 h by the addition of isopropyl-β-d-thiogalactopyranoside (IPTG) to a final concentration of 0.1 mM. Next, the culture was harvested by centrifugation (5000× *g*, 20 min, 4 °C) and the pellet was resuspended in lysis buffer (300 mM NaCl, 20 mM Tris-HCl, 10 mM imidazole, 5% (*v/v*) glycerol, pH 7.8) containing complete protease inhibitor cocktail (Roche Diagnostics, Mannheim, Germany). After sonication, the whole-cell lysate was then centrifuged (12,500× *g*, 30 min, 4 °C) and the soluble fraction was filtered through a 0.22 µm filter (Millipore, Darmstadt, Germany). The His-tagged protein purification was performed with a Ni-NTA His∙Bind^®^ Resins (Novagen, EMD Millipore, Darmstadt, Germany) in gravity columns or Bio-Scale Mini Profinity IMAC cartridges (Bio-Rad, Hercules, CA, USA) in combination with FPLC-system (Bio-Rad). After column equilibration with the washing buffer (300 mM NaCl, 20 mM Tris-HCl, 10 mM imidazole, 5% (*v/v*) glycerol, pH 7.8), the lysate was loaded and washed with 15 volumes of washing buffer. The protein was eluted (300 mM NaCl, 20 mM Tris-HCl, 150 mM imidazole, 5% (*v/v*) glycerol, pH 7.8) and dialyzed overnight at 4 °C against a 1000-fold volume of phosphate-buffered saline (PBS) buffer (137 mM NaCl, 2.7 mM KCl, 10 mM Na_2_HPO_4_, 1.8 mM KH_2_PO_4_, pH 7.4) using Float-A-Lyzer (Serva, Heilderberg, Germany). Affinity-purified protein was concentrated to 1.5 mg/mL by centrifugation over a 30-kDa cut-off membrane (Amicon Ultra Centrifugal Filters, Merck Millipore, Darmstadt, Germany). A 0.5 mL sample was loaded onto a Superdex 200 10/300 column (GE Healthcare, Little Chalfont, UK), equilibrated, and eluted with a buffer containing 150 mM NaCl, 20 mM Tris-HCl, 2% (*v/v*) glycerol pH 7.8 with a constant flow rate of 0.5 mL/min.

### 2.3. Protein Analysis Assays

Sodium dodecyl sulfate-polyacrylamide gel electrophoresis (SDS-PAGE) was performed according to the method of Laemmli [24] using a 12% polyacrylamide gel or precast Tris-HCl PAGE gels (4%–20%, gradient polyacrylamide gels, Bio-Rad), after heating the protein samples at 95 °C for 5 min. To evaluate enzyme susceptibility to SDS and proteolysis, the samples of 30 μL containing 250 μg/mL depoKP36 in the buffer (50 mM Na_2_HPO_4_, pH 7.0) containing 1% SDS (Bio-Rad) or 1% trypsin (Gibco BRL, Paisley, UK) were incubated for 10 min at room temperature (RT) or 100 °C and at 37 °C for 1 h, respectively. Further, the protein samples were mixed with Laemmli buffer (Bio-Rad), and analyzed in SDS-PAGE as heated and unheated samples. For native PAGE, TGX precast gels (Bio-Rad), and running buffer (25 mM Tris, 192 mM glycine, pH 8.3) were used. Sample buffer (62.5 mM Tris-HCl, 25% glycerol, 1% bromophenol blue, pH 6.8) was mixed with a protein sample (1:1 *v/v*) and loaded without heating. Molecular weight (MW) standards (10–250 kDa or 6.5–212 kDa) were used (Bio-Rad). The protein bands were visualized by Coomassie brilliant blue R-250 (Bio-Rad) staining.

For zymogram assays, samples were boiled for 5 min before applying them to a 12% SDS-PAGE gel, which contained 45% (*v/v*) crude EPS from *K. pneumoniae*. After electrophoresis, the zymograms were washed with MilliQ water at RT and transferred to a buffer (0.15 M NaH_2_PO_4_, 10 mM MgCl_2_, and 0.1% (*w/v*) Triton X-100; pH 7.0) for 48 h at RT for in situ protein renaturation. Following this, gels were rinsed with MilliQ water, stained with methylene blue (0.1% (*v/v*), 0.001% (*w/v*) KOH) for 12 h at RT, and then destained with water until enzyme activity bands appeared.

The protein concentration was measured fluorometrically (Qubit 2.0, Thermo Fisher Scientific) or spectrophotometrically (NanoDrop 2000 Spectrophotometer, Thermo Fisher Scientific) using a molar extinction coefficient of 94,240 cm^−1^∙M^−1^ calculated by ProtParam program [25].

### 2.4. Extraction and Purification of Exopolysaccharide (EPS)

To extract and purify EPS, a crude EPS fraction was prepared from supernatants of five-day *K. pneumoniae* 486 cultures, according to the method of Bales et al. [26]. Briefly, 200 mL of TSB was inoculated with 20 mL of an overnight culture of *K. pneumoniae* and incubated at 37 °C without agitation for 5 days. Following the addition of 60 µL formaldehyde (36.5% solution) to each 10 mL of culture for 1 h and then 1 M NaOH for 3 h at RT, with agitation, the secreted EPS was separated from bacterial cells by centrifugation (16,800× *g*, 1 h, 4 °C). The proteins and nucleic acids were precipitated by adding of trichloroacetic acid (TCA; 20% *w/v*) and subsequently centrifuging the solution (16,000× *g*, 1 h, 4 °C). The pellet was discarded and EPS remaining in the supernatants was precipitated away from lipids by adding 1.5 volumes of 96% cold ethanol at −20 °C for 24 h. The precipitate was collected by centrifugation (16,800× *g*, 1 h, 4 °C) and resuspended in MilliQ water. The crude EPS was dialyzed against an excess of MilliQ water for 24 h at 4 °C using a 12–14 kDa molecular weight cut-off (MWCO) membrane (Serva, Heidelberg, Germany) to remove low molecular weight impurities, and the remaining retentate was lyophilized overnight. Next, the lyophilized powder was resuspended in 5–10 mL of water to a final concentration of 4 mg/mL.

### 2.5. Functional Assays

To visualize the enzyme activity, identifying the sensitivity of *Klebsiella* strains and comparing it to phage-mediated lysis, spot assay was used. For this purpose, log-phase bacteria were transferred directly onto TSA plates. After drying, both 10 µL of serially diluted recombinant enzyme or 10 µL the phage suspension (10^8^ PFU/mL) was spotted onto the bacterial lawn. After overnight incubation at 37 °C, plates were observed for formation of clear zones (halo) or lytic zones (plaque) surrounded by a halo, respectively. For determination of bacterial count both in the halo zone and bacterial lawn, at least two equal square agar blocks (5 mm × 5 mm) were cut out from each zone from three different plates. Each block was suspended in 1 mL of PBS and vortexed. The number of bacteria was counted after plating a dilution series of the supernatant. Bacterial cell viability after depoKP36 treatment was also evaluated. Briefly, the log-phase bacteria (~2 × 10^8^ CFU/mL) were incubated with the enzyme (final concentration 280 μg/mL) for 2 h at 37 °C. The number of viable bacteria were determined at the beginning and, afterwards, exposed to enzyme by serial dilution in PBS followed by overnight growth at 37 °C on TSA plates.

The activity of depoKP36 at various pH levels (ranging from 3.0 to 9.0) and at various temperatures (ranging from 20–80 °C) was assessed by measuring the decrease in turbidity according to the method reported by Belleman et al. [27] with some modifications. Briefly, aqueous EPS solution extracted from *K. pneumoniae* 486 (4 mg/mL), was dissolved in an appropriate buffer to final concentration 0.5 mg/mL. The enzyme reaction was initiated by adding 115 µL of enzyme to 996 µL of substrate solution. The mixture was incubated for 30 min at the desired buffer or temperature and then cooled. Remaining polysaccharides were precipitated by adding cetylpyridinium chloride (CPC; Sigma-Aldrich, St. Louis, MO, USA) to a final concentration of 5.1 mg/mL. After 10 min incubation at RT, the absorbance at 600 nm was measured (Asys UVM340, Biochrom Ltd., Cambourne, UK). Relative enzyme activity was calculated and was expressed as percent reduction of turbidity compared with control without enzyme. The influence of pH on enzyme activity was determined at 20 °C using the following buffers: 50 mM sodium acetate buffer (pH 3.0–5.0), 50 mM Na_2_HPO_4_ (pH 6.0–7.0), and 50 mM Tris-HCl buffer (pH 8.0–9.0). To study the effect of temperature on enzyme activity, assays were performed in 50 mM sodium acetate buffer (pH 5.0) in the range 20–80 °C. For the measurement of time evolution of enzyme activity at different temperatures, depoKP36 was pre-incubated at the desired temperature in buffer at pH 5.0 and samples were taken at regular time intervals (0, 10, 20, 30, 40, 50, and 60 min). After cooling the samples, residual activity was determined at 20 °C using the standard protocol described above. Each experiment was performed in quadruplicate and repeated at least twice.

### 2.6. Galleria Mellonella Larvae Infection Model

Wax moth larvae (*G. mellonella*) were obtained from a culture of insects. Briefly, the larvae were reared on a natural diet—honeybee nest debris at 30 °C in the dark. Prior to inoculation, the last instar larvae were selected to be similar in size (approximately 250–350 mg) and were then maintained on wood chips in darkness at 15 °C for 3–4 days. Bacteria from overnight culture were grown in TSB at 37 °C to log-phase for 2 h, harvested (5000× *g*, 20 min, 4 °C), washed with PBS and suspended in PBS to an optical density at 600 nm of 1.0 corresponding to ~10^9^ CFU/mL. Larvae were inoculated with 10 μL of bacterial suspension containing 10^7^ CFUs into the last pro-leg using insulin syringe with a 30-gauge needle (NIO Lab, Nieborow, Poland). The anti-virulence effect of enzyme on *K. pneumoniae* 486 was estimated by inoculated larvae with either bacteria pretreated with depoKP36 (final concentration, 280 μg/mL) for 2 h at 37 °C, or enzyme administered within 5 min after untreated bacterial infection. Three control groups were used: uninfected larvae, larvae injected with PBS to monitor the killing due to injection trauma, and larvae injected with depoKP36 to assess the toxicity of the enzyme. After inoculation, caterpillars were kept at 37 °C in the dark for 72 h. The results were expressed as the percentage survival rate estimated on the basis the touch-provoked motility and the appearance of pigmentation at 24 h, 48 h, and 72 h post injection. For each option, at least three independent experiments were performed (10 larvae per trial). Survival curves were plotted using the Kaplan-Meier method, and the analysis in survival was performed by using the log-rank Mantel-Cox (GraphPad Software, Inc., La Jolla, USA). A *p*-value of < 0.05 was considered to be statistically significant.

### 2.7. Antimicrobial Susceptibility Testing

*K. pneumoniae* strains (486 and 77) susceptibility to the antibiotics representing four different classes: aminoglycosides (gentamicin sulfate; MP Biomedicals, Eschwege, Germany), fluoroquinolones (ciprofloxacin; MP Biomedicals, Eschwege, Germany), tetracyclines (oksytetracycline; MP Biomedicals, Eschwege, Germany), and chloramphenicol (Calbiochem, Darmstadt, Germany), was determined by a broth microdilution technique according to Clinical and Laboratory Standards Institute (CLSI) recommendations [28]. For quality control, *K. pneumoniae* ATCC 700603 was included in each set of tests. The minimal inhibitory concentration (MIC) was determined as the lowest antibiotic concentration at which there is no visible growth.

To evaluate the activity of depoKP36 combined with antibiotics against *K. pneumoniae* strains, the checkerboard broth microdilution method was used. Two-fold serial dilutions of the antibiotic, in a range between 2 MIC and 1/4 MIC, and two-fold serial dilutions of the enzyme (final concentrations from 125 μg/mL to 7.81 μg/mL) were prepared for every option tested and 100 µL aliquots of each component was added into the wells of the sterile 96-well microtiter plate (VWR International, Darmstadt, Germany), along the ordinary and abscissa, respectively. Then each well was inoculated with 10 μL of a bacterial inoculum of 5 × 10^5^ CFU/mL, and the plates were incubated at 37 °C for 18 h. The MIC was determined as the lowest concentrations of antibiotic in combination with the enzyme giving complete inhibition of visible growth.

### 2.8. Light Scattering Experiments

Purified, not aggregated depoKP36 was analyzed by size-exclusion chromatography (SEC) coupled to a miniDAWN TREOS multi-angle static light scattering (MALS) detector (Wyatt Instrument Technology Corp., Santa Barbara, CA, USA) and an OptilabTM rEX (Wyatt Instrument Technology Corp.). The protein sample of 2 mg was loaded on a S200 10/30 column, previously equilibrated in 200 mM NaCl, 25 mM HEPES, 5% glycerol, pH 7.8. A constant flow rate of 0.5 mL/min was applied. The online measurement of the intensity of the Rayleigh scattering as a function of the angle as well as the differential refractive index of the eluting peak in SEC was used to determine the average MW of eluted protein using Astra 5.3.4.14 (Wyatt Instrument Technology Corp.) software.

### 2.9. Circular Dichroism (CD) Studies

Circular dichroism (CD) spectroscopy and thermal melting curves were recorded on a Jasco J-810 spectropolarimeter fitted with a single cell Peltier temperature controller (Model PTC-423S). CD measurements were carried out at 20 °C in a 0.1 cm optical path length cell with protein concentration of 0.2 mg/mL in 20 mM sodium acetate buffer (pH 6.0) containing 150 mM NaCl. Each spectrum was scanned from 260 nm to 190 nm with an integration time of 3 s at each wavelength. All spectra were averaged from three scans and baseline-corrected using a blank consisting of sodium acetate buffer (20 mM, pH 6.0). The molar ellipticity per mean residue, [Ѳ] in deg·cm^2^·dmol^−1^, was calculated from the following equation: [Ѳ] = [Ѳ]obs × mrw × (10 × l × C)^−1^ where [Ѳ]obs is the ellipticity measured in degrees, mrw is the mean residue molecular mass (106.5 Da), l is the optical path length of the cell in cm, and C is the protein concentration in g/L. Thermal denaturation studies were conducted at 214 nm with increasing temperature from 20 °C to 90 °C. The protein solution was equilibrated at each temperature point for 2 min, and the temperature was increased with an average rate of 0.5 °C/min. The melting temperature (T*m*) was obtained by taking the peak of the first derivative of the melting curve. The data were then normalized, and the T*m* was inferred from the loss of 50% of the maximum signal.

## 3. Results

### 3.1. Identification of Phage KP36 gp50 as a Putative EPS Depolymerase

The ability of bacteriophages to enzymatically degrade bacterial polysaccharides is an effective strategy allowing the virions to deliver their genome across the bacterial envelope to the cell cytoplasm, where their genetic information is expressed and replicated. While studying the phage KP36, we noticed the appearance of hazy, expanding halo zones surrounding plaques with a small clear center formed by it on a bacterial lawn [21]. This observation indicated that phage KP36 may produce a virion-associated polysaccharide-degrading enzyme.

**Figure 1:**
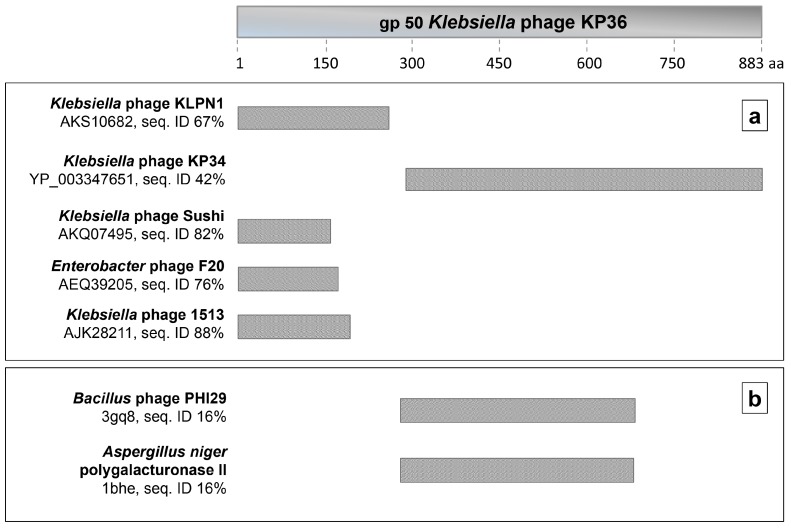
Bioinformatic analysis of *Klebsiella* phage KP36. (**a**) BlastP analysis in non-redundant sequence database; (**b**) HHPred analysis in Protein Data Bank (PDB).

Protein Blast (BlastP) analysis of the *gp50* gene product (putative tailspike protein) showed high sequence identity of the N-terminal region to the tail fiber protein of *Klebsiella* phage Sushi and hypothetical phage proteins of *Klebsiella* phage 1513, *Enterobacter* phage F20 and *Klebsiella* phage KLPN1 (Figure 1a) [29]. Moreover, the C-terminal region has significant sequence identity to *Klebsiella* phage KP34 gp57. Amino acid sequence also evidenced a fragment exhibiting similarity to the pectate lyase superfamily (residues 263–330). Of note, none of the aforementioned phage proteins has yet been characterized in vitro.

Whereas no significant sequence similarity with structurally determined proteins was found using BlastP, sequence comparisons using consensus sequence methods by HHPred identified two moderate homologs in the Protein Data Bank (PDB) database [30]. Those included a pre-neck appendage protein from *Bacillus* phage phi29 (pdb code 3gq8) and polygalacturonase II from *Aspergillus niger* (pdb code 1bhe), (Figure 1b). Both proteins contain high β-helical structure. Consistently, secondary structure predictions using PSIPRED [31] indicate that gp50 of phage KP36 possesses a dominant amount of beta-structure (53%), similar to other identified polysaccharide binding enzymes of viral or microbial origins [32,33].

All of this information suggested that the product of gene *gp50* is a probable polysaccharide depolymerase involved in the phage attack against its host bacterium. Therefore, we cloned, expressed, and purified the full-length protein (883 amino acids) with predicted molecular mass of 93.4 kDa and a pI of 5.0, here designated as depoKP36, to gather understanding on its functional and structural properties.

### 3.2. Recombinant depolymerase enzyme encoded by Klebsiella phage KP36 (depoKP36) Degrades Bacterial EPS and Shows a Narrow Spectrum Activity Against Specific *K. pneumoniae* Strains

**Figure 2:**
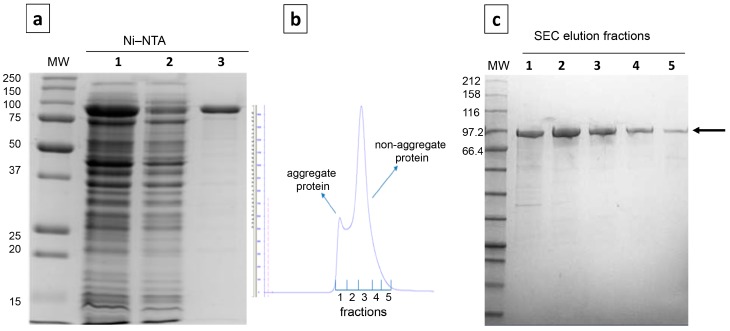
Expression and purification of the depolymerase enzyme encoded by *Klebsiella* phage KP36 (depoKP36); (**a**) Ni-NTA His∙Bind® Resins affinity column purification. Protein samples run on a reducing 12% gel. Lane MW: molecular weight (MW) markers; lane 1: lysate of induced *E. coli* BL21 pEXP-5-CT/TOPO^®^-depoKP36 cells; lane 2: column wash with buffer containing 10 mM imidazole; lane 3: eluted proteins; (**b**) Gel filtration chromatogram; (**c**) Eluted protein after gel filtration chromatography. Protein samples were separated on 4–20% sodium dodecyl sulfate-polyacrylamide gel electrophoresis (SDS-PAGE) gel. Lane MW: MW markers; lane 1: aggregate protein; lanes 2, 3, 4, 5: non-aggregate protein fractions. Samples were stained using Coomassie brilliant blue R-250. The arrow indicates protein of interest, depoKP36; SEC: size-exclusion chromatography.

The recombinant depoKP36 was purified in the two-step strategy coupling nickel affinity and gel filtration procedures (Figure 2). The size-exclusion chromatography profile indicated that the purified protein was predominantly homogeneous, with only a small proportion in an aggregated state. Only fractions involving the main peak corresponding non-aggregated protein were pooled and subjected to further studies. The purified depoKP36 migrated as a single band on SDS-PAGE corresponding to an approximately 93.4 kDa protein.

**Figure 3:**
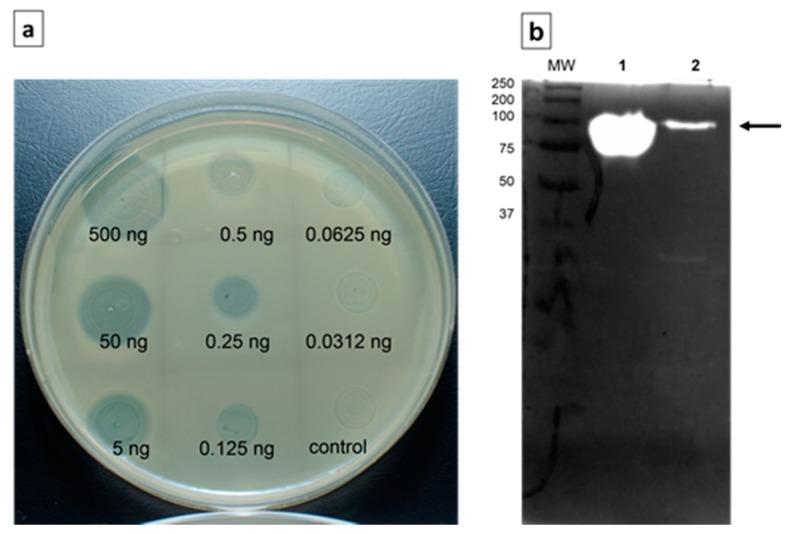
The activity of depoKP36 for its natural substrate. (**a**) Spot test with serial dilutions of depoKP36 on host strain. Phosphate-buffered saline (PBS) was used as a control; (**b**) Zymography. Protein samples run on a 12% standard Laemmli SDS-PAGE gel containing crude exopolysaccharide (EPS) as a substrate. Zymograms were stained with methylene blue. Lane MW: MW markers; lane 1: recombinant depoKP36 (2.5 μg/lane); lane 2: phage KP36 (10^9^ PFU/lane).

The activity of depoKP36 was tested on *K. pneumoniae* 486 lawn by spot assay with halo zone detection using enzyme dilutions ranging from 50 μg/mL to 3.1 μg/mL (Figure 3a). The capsular staining of bacteria present in and outside of zone indicated the degradation of capsular material. The presence of almost equal amounts of viable cells in samples from both areas (2.9 × 10^9^ ± 3.0 × 10^8^ CFU/mL and 3.6 × 10^9^ ± 3.5 × 10^8^ CFU/mL, respectively) suggested the lack of affected cell viability.

To confirm that depoKP36 acts as polysaccharide depolymerase, we subsequently extracted and purified EPS from supernatants of host culture and applied zymography analysis to visualize its enzymatic activity. As shown in Figure 3b, a single band of clearing at ~94 kDa, which corresponds to the size of the complete depoKP36 protein, was observed on zymograms. The same band was seen after phage KP36 application.

Enzymatic activity of depoKP36 was further tested on clinical *K. pneumoniae* strains panel using spot test. The phage KP36 was included as a positive control. Among the 11 strains tested, eight (73%) isolates corresponded to the K63 reference strain, the others represented the three other capsular types, namely K3, K10 and K20. Only K63 type bacteria proved to be susceptible to CPS degradation by both the protein and phage, whereas none of the other aforementioned capsular types were as sensitive.

### 3.3. Anti-Virulence Efficacy of depoKP36 in the G. mellonella Infection Model

**Figure 4:**
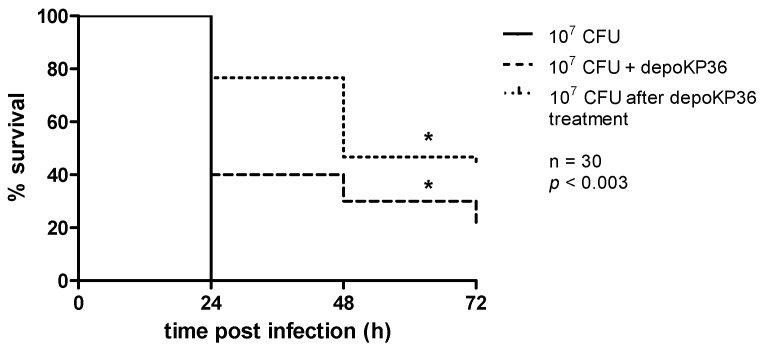
Inhibition of *K. pneumoniae*–induced mortality by depoKP36 using *G. mellonella* model. Larvae (*n* = 30) were injected with either bacteria (10^7^ CFUs/per larvae), enzyme (final concentration, 280 μg/mL) administered within 5 min after untreated bacteria inoculation, or depoKP36-treated bacteria, and monitored for mortality. The experiments were controlled by observation of uninfected larvae, PBS-injected larvae and larvae receiving the enzyme only. Survival for each control group was 100%, so for simplicity, these groups were not included in the figure. Survival curves were plotted using the Kaplan-Meier method, and differences in survival were calculated by using the log-rank test (GraphPad); * *p* < 0.003 (considered to be statistically significant); Results are the means of at least three independent experiments.

Given the therapeutic efficacy of depolymerase in murine model of *K. pneumoniae* infections [17] and the capsule necessity for *K. pneumoniae* virulence in *G. mellonella* larvae [34], we evaluated the virulence of depoKP36-treated *Klebsiella* strain as well as the efficacy of treatment with this enzyme in vivo using the insect model. Without the treatment, 100% of the larvae died within 24 h after inoculation of 10^7^ cells of *K. pneumoniae* 486 (Figure 4). In contrast, a single dose of enzyme given within 5 min after bacteria injection as well as inoculation of bacteria pretreated with depoKP36 for 2 h, significantly inhibited *K. pneumoniae*-induced death in a time-dependent manner (*p* < 0.003). At 24 h and 48 h post application of depoKP36, the survival rate of *Galleria* increased by up to 40% and 30%, respectively. At 72 h another 10% of the remaining survivors were dead. In turn, bacteria treated with depoKP36 prior to inoculation killed only 23% of the larvae at 24 h compared to 53% and 57% mortality at 48 h and 72 h, respectively. No mortality of larvae was observed in the controls, upon injection of PBS buffer or depoKP36. No differences in the number of viable bacteria were detected before (2.58 × 10^8^ CFU/mL ± 5.12 × 10^7^ CFU/mL) and 2 h after exposure to depoKP36 (2.8 × 10^8^ CFU/mL ± 4.07 × 10^7^ CFU/mL).

### 3.4. DepoKP36 Do Not Affect the Action of Antibiotics Against K. pneumoniae Strains

To gain further insight of the therapeutic potential of depoKP36, we evaluated the activity of the enzyme in combination with four classes of antibiotics against two *K. pneumoniae* strains (486 and 77) representing the K63 capsular type. The MIC values have not changed for each particular antibiotic after depoKP36 application indicating that depolymerase did not alter the potency of any antibiotics tested. The ciprofloxacin, oxytetracycline, and chloramphenicol MICs for both *Klebsiella* strains were 0.125–0.25 μg/mL, 1 μg/mL, and 2μg/mL, respectively, whereas the MIC of gentamicin was 0.5 μg/mL for strain 486 and >32 μg/mL for strain 77.

### 3.5. DepoKP36 Remains Stable at Moderately Acidic Conditions and Is Mesophilic

**Figure 5:**
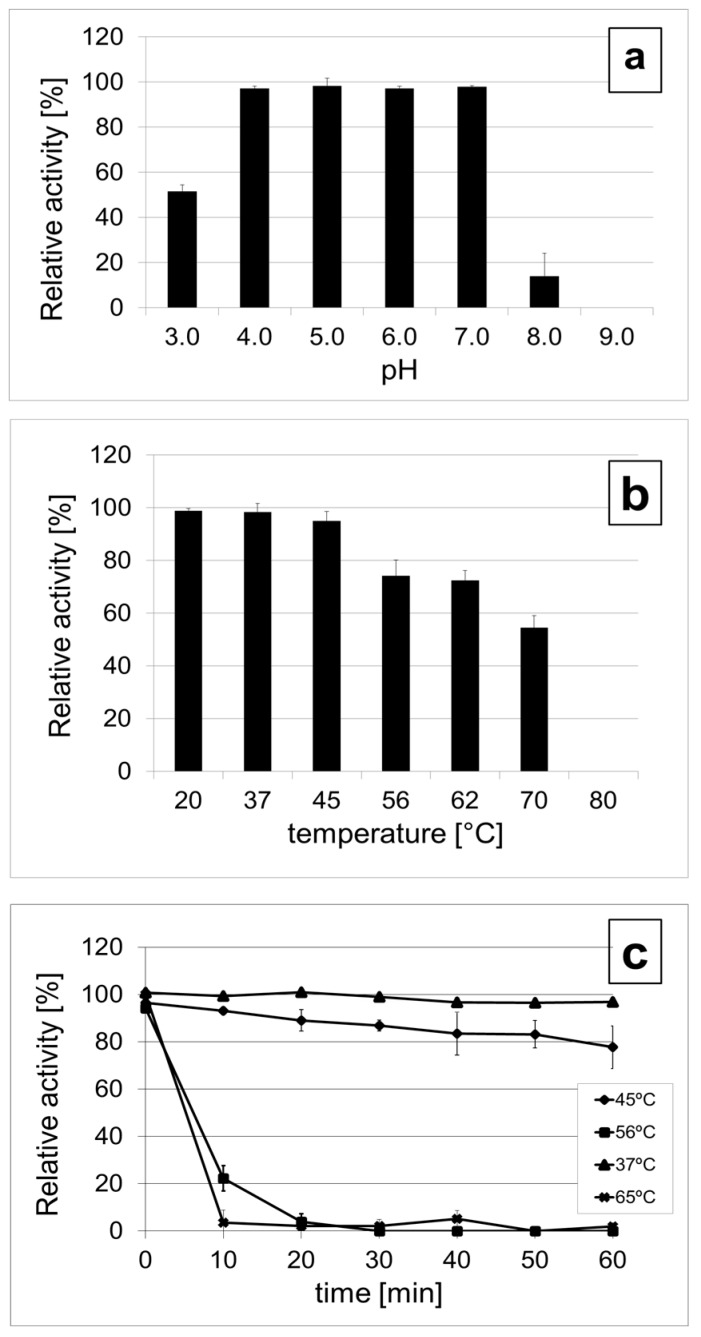
EPS-degrading activity of depoKP36. (**a**) Effect of pH on the activity of depoKP36. The optimal pH was determined at 37 °C in 50 mM CH_3_COONa-HCl buffer (pH 3.0–5.0), 50 mM Na_2_HPO_4_ buffer (pH 6.0–7.0), and 50 mM Tris-HCl buffer (pH 8.0–9.0); (**b**) Influence of various temperatures on the activity of depoKP36 at pH 5.0; (**c**) Time evolution of depoKP36 activity at 37 °C, 45 °C, 56 °C and 65 °C. Enzyme was pre-incubated in the absence substrate for 10, 20, 30, 40, 50, and 60 min at desired temperature before measuring its activity. Relative enzyme activity was calculated and is expressed as a percent reduction of turbidity compared with control without depoKP36. Each experiment was performed in quadruplicate and repeated at least twice. The data represent means ± standard deviation (SD).

A quantitative measure of the enzymatic activity of depoKP36 in a range of pH and temperatures was obtained by monitoring the decrease of turbidity of aqueous solutions of EPS extracted from *K. pneumoniae* 486 (0.5 mg/mL) following the enzyme action (Figure 5). Relative enzyme activity was expressed as percent reduction of turbidity compared with the control without enzyme. The activity range of depoKP36 on its natural substrate as a function of either pH or temperature was determined after 30 min incubation in the proper buffer. As a result, we observed that depoKP36 remains active over a pH range, from 4.0 to 7.0, with relative activities ranging from 97.1% ± 1.02% to 97.8% ± 0.57%. The activity diminished substantially as the pH was lowered below 4.0 or raised above 7.0, to 54.6% ± 2.81% and 14.0% ± 14.17% of initial activity, respectively. Based on these results, depoKP36 can be considered an acidically stable enzyme. Therefore, the enzyme activity at pH 5.0 in the temperature range from 20 °C to 80 °C was investigated. Optimal activity of the enzyme was found at temperatures ranging from 20 °C to 37 °C, which probably reflect adaptation to the physiological environment of the *K. pneumoniae* bacteria. Non-significant reduction, to 95% of the initial activity, was observed at 45 °C, whereas heat treatments at 56 °C and 62 °C lowered the enzyme relative activity to 70%. When heated at 70 °C, the enzymatic activity of depoKP36 fell below 60% of the initial activity. At higher temperatures, depoKP36 was completely inactivated.

To gain further insight into the effect of temperature on enzyme activity in solution, depoKP36 was preincubated without the substrate at different temperatures (from 37 °C to 65 °C) in a buffer at pH 5.0, and the samples were drawn at various time points. The incubation at 37 °C has no effect on relative turbidity whereas time evolution of turbidity measurements at 45 °C shows a small decrease (77.6% ± 9.0%) after 60 min of incubation. The decrease of turbidity becomes drastic at 56 °C and 65 °C, with complete suppression after 30 and 10 min, respectively. Altogether, these data show that depoKP36 is active in a wide range of temperatures, from 20 °C to 45 °C.

### 3.6. Structural Characterization of depoKP36 in Solution

**Figure 6:**
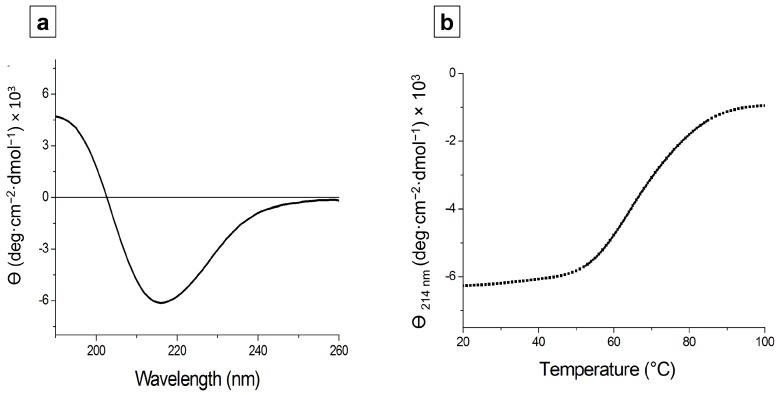
(**a**) Circular dichroism (CD) spectrum of depoKP36 (0.2 mg/mL) in sodium acetate 20 mM, pH 6.0; (**b**) Thermal denaturation curve measured at 214 nm. The midpoint of the curve was used to calculate the melting transition temperatures (T*m*) for protein.

A search of the PDB database identifies polygalacturonase II from *Aspergillus niger* (seq id 13% on residues 206–383) as the protein of known structure most similar to depoKP36. Due to this scarce available structural information, we carried out structural studies in solution using CD and light scattering experiments. CD spectra showed that depoKP36 adopts a well-folded conformation, with a negative dichroic minimum between 210 and 220 nm and a positive dichroic maximum between 195 and 200 nm, characteristic of a protein with high β-sheet content (Figure 6a). To investigate the heat-induced changes in the protein secondary structure, thermal unfolding curves were achieved by following the CD signal at 214 nm as a function of temperature. Thermal unfolding curves show a sigmoidal transition with melting temperature (T*m*) = 65 °C (Figure 6b).

**Figure 7:**
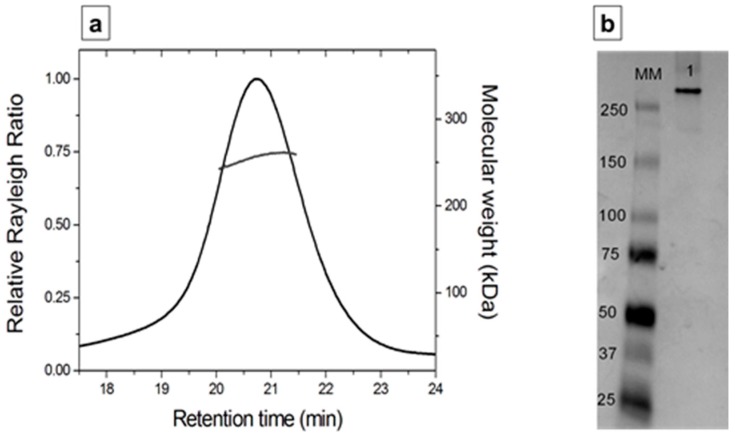
(**a**) Analytical SEC, coupled with multi-angle static light scattering (MALS) of depoKP36. The black curve represents the Rayleigh ratio (left scale) against the retention time. Molecular mass (right scale) values correspond to a trimeric state; (**b**) Native PAGE electrophoresis of depoKP36. Lane MM: molecular weight markers, lane 1: recombinant depoKP36.

Analytical SEC, coupled with MALS (SEC-MALS), was carried out to investigate the oligomerization state of depoKP36 in solution. The online measurement of the intensity of the Rayleigh scattering as a function of the angle as well as the differential refractive index of the eluting peak in SEC was used to determine the MW (Figure 7a). This analysis produced an MW of 255 kDa, which corresponds to a trimeric organization of the protein in solution. This MW was also confirmed by native gel electrophoresis (Figure 7b).

**Figure 8:**
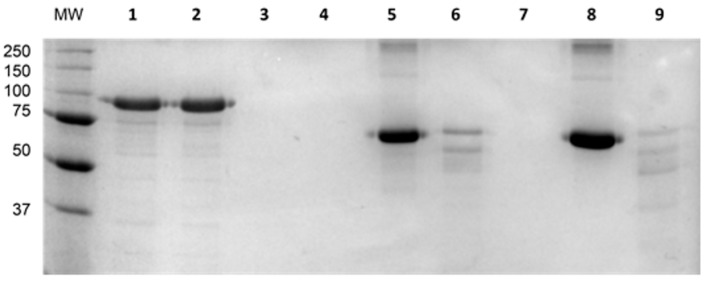
Susceptibility of depoKP36 to denaturation in the presence of 1% SDS and proteolysis. Lane MW: molecular weight markers; lanes: (**1**) depoKP36 boiled, (**2**) depoKP36 non-boiled, (**3**) depoKP36 + trypsin, boiled, (**4**) depoKP36 + trypsin, non-boiled, (**5**) BSA boiled, (**6**) BSA + trypsin, boiled, (**7**) trypsin boiled, (**8**) BSA non-boiled, (**9**) BSA + trypsin, non-boiled.

Furthermore, to analyze the structural integrity of depoKP36 in the presence of both anionic detergent and protease, the electrophoretic mobility of the enzyme was studied. As shown in Figure 8, depoKP36 migrated as a monomer at 94 kDa during SDS-PAGE regardless of SDS-treated sample heating, which indicates that SDS is sufficient for enzyme denaturation. In turn, the lack of a visible band reflecting this protein on the gel after trypsin treatment suggests its vulnerability to proteolytic cleavage.

## 4. Discussion

Given the crucial role of the CPS in the pathogenesis of *Klebsiella* infections, it is not surprising that the potential of phage-encoded proteins with polysaccharide depolymerization activity to generate “capsule-stripped” strains has been the subject of extensive studies [17,20,35,36,37,38]. Such enzymes can sensitize the bacterial cells making them more susceptible to a component of the host’s immune system as well as to antimicrobials [39,40]. The other advantages of these proteins are the low incidence of resistance generation and lack of side effects on normal microbiota [40,41]. For these reasons, a novel depolymerase enzyme, encoded by *Klebsiella* phage KP36 from the *Siphoviridae* family, has been identified and characterized in this study to evaluate its effectiveness against encapsulated bacteria in vitro and in vivo. As other recombinant enzymes that cleave *Klebsiella* CPS reported so far, were encoded by *Podoviridae* and *Myoviridae* phages [8,20,36,38,42], depoKP36 constitutes the first recombinant depolymerase obtained from siphovirus specific against *Klebsiella*.

NCBI BLAST results revealed that depoKP36 shares about 67–88% amino-acid identities in the N-terminal region with other putative tail fiber proteins from *Klebsiella* and *Enterobacter* phages, belonging to the same family of *Siphoviridae* and more specifically the “*KP36likevirus*” genus [22,43]. Such high homology within morphologically similar phages may indicate the presence in this part of a domain anchoring the tail fiber to the phage particle. In turn, the exhibition of a C-terminal sequence similarity to the gp57 of *Klebsiella* phage KP34 from *Podoviridae*, and the identification of common host spectrum for both phages [21,44] suggests that it is the region responsible for recognizing polysaccharides and binding to the host cell receptor. Also, a pectate lyase domain fragment, found in the sequence alignment, indicates the involvement of depoKP36 in the modification or cleavage of glycoside bonds in the capsular polysaccharide. Previously, this domain has been reported in other phage tail spike proteins targeting and degrading CPS, like K5 lyase of coliphage K5A [45] and Tsp2 of phage ΦSH19 [46].

Our study proved that depoKP36 is able to digest the capsule from the surface of all isolates with a wzi sequence corresponding to the K63 reference strain. Also, the zymography confirmed that this enzyme efficiently degrades purified EPS from *Klebsiella* serotype 63. Notably, this polysaccharide structure with repeating units of [→3)-α-d-Galp-(1→3)-α-d-GalpA-(1→3)-α-l-Fucp (1→] [47] is identical to that of *E. coli* serotype K42 capsular polysaccharide [48], a finding which could potentially extend the spectrum of bacteria susceptible to this protein. Furthermore, modifications of depolymerase could further expand its bacterial spectrum [49]. Indeed, it has been shown that even a single amino acid substitution in the active site of phage HK620 tailspike protein results in an increase of binding affinity of up to three orders of magnitude [50] and that, upon mutations, the enzyme can gain the ability to recognize other bacterial surface receptors [51].

Together with the evaluation of the ability of depoKP36 to degrade capsular polysaccharides in vitro, we investigated its effect in vivo using the *G. mellonella* model. The innate immune response of insects in many aspects reflects the defensive mechanisms against *Klebsiella*-triggered pneumonia in animals [34]. In our study, the results obtained on the wax moth larvae model confirm the idea of utilization of these phage-encode proteins as anti-virulent agents. The increased larval survival rates following the application of the enzyme or bacteria treated with it prior to inoculation suggests the decrease in bacterial virulence as the consequence of the loss of capsule layer as the major *Klebsiella* pathogenicity factor.

Given the improved gentamicin efficacy against *K. pneumoniae* serotype K2 after using the bacterial depolymerase [40], we further evaluated the activity of depoKP36 in combination with four classes of antibiotics. Unlike the in vivo studies involving the bacterial enzyme [40], our in vitro experiments have not revealed the ability of phage-derived depolymerase to potentiate the efficacy of aminoglycoside antibiotic against *K. pneumoniae*. Also, the efficacy of ciprofloxacin (fluorochinolones), oksytetracycline (tetracyclines), and chloramphenicol did not change after administration of depoKP36. Nevertheless, besides no improvement of antibiotic activity it is worth mentioning that depolymerase treatment does not disturb drug action. Thus, reducing other virulence factors responsible for the susceptibility to immune response, the final result of depolymerase application may lead to the improvement of the drug/enzyme combined therapy.

Our results showed that depoKP36 displays moderate acidic-stability with the optimum pH in the range from 4.0 to 7.0, similar to the depolymerase from *Klebsiella* phage P13 [38]. This pH-dependence of enzyme activity agrees well with the ability of *K. pneumoniae* to infect the urinary tracts, as the pH of urine ranges from 6.5 to 7.0. From this perspective, such enzymes could be interesting candidates for anti-virulence agents in a hurdle approach for catheter preservation, in addition to other antimicrobial additives. On the contrary, the enzymes from phages specific to *K. pneumoniae* 390 and B5055 were found to be stable in a pH range shifted toward more alkaline conditions, from 6.0 to 9.0 or 10.0, respectively [14,36]. The activity of depoKP36 was optimal in the range from 20 °C to 37 °C, what ensures the best efficacy during the therapy, as well as anti-virulent/anti-biofilm preservation agent when applied as catheter additive. The trimeric structure of depoKP36, as reported for other tailspike proteins, including those from phages: P22 [52], Sf6 [53], HK620 [54], phi 29 [55], Det7 [56], and 9NA [57], provides a high stability at different pH and temperature conditions, but most importantly it increases the avidity of protein-carbohydrate interactions [49]. Unlike most of the described viral fibers, the depoKP36 remains susceptible to SDS-induced denaturation and proteolytic cleavage [33,58]. In proteins with a high content of β-structure, denaturation in the presence of SDS increases the amount of α-helix structure [59]. This favors the formation of a “bead-on-a-string” structure, in which the SDS micelles bind to proteins, stabilizing it in this way [60]. This also suggests that SDS-conformational changes might facilitate access of the active site of the enzyme to the substrate.

In conclusion, the identification of a novel depolymerase and the characterization of its catalytic and structural features add insights to the understanding of the mode of action of phage-derived enzymes able to digest bacterial polysaccharides. Altogether, our findings demonstrate that depoKP36 is the enzyme responsible for the ability of KP36 phage to degrade the bacterial capsule, making bacteria less pathogenic and more easy to eradicate by immune response mechanisms. What is more, the bacterial capsule serves as the effective adherence element conditioning the formation of biofilm structure on living and artificial surfaces. Therefore, our results provide grounds to use depoKP36 as a phage-based anti-biofilm tool. Indeed, the observed efficacy, stability, and specificity of depoKP36 makes it suitable for various purposes, including the monitoring of biofilm formation and bacterial detection, coating of medical tools and devices, and glycans analyses.
